# Spontaneous urinary bladder rupture in a dog with lymphoplasmacytic cystitis

**DOI:** 10.1111/jsap.13858

**Published:** 2025-03-26

**Authors:** C. Donà, M. Manfredi, L. Auletta, M. Zambelli, E. Brambilla, J. Bassi, M. Longo

**Affiliations:** ^1^ Department of Veterinary Medicine and Animal Sciences (DIVAS) University of Milan Lodi Italy; ^2^ Zooprophylactic Institute of Lombardy and Emilia‐Romagna Brescia Italy

## Abstract

A 10‐year‐old male mixed‐breed dog presented with vomiting and anuria. The dog was living indoors, and no trauma was reported by the owner. Ultrasonography and a retrograde urethrogram revealed the presence of a urinary bladder leakage. A celiotomy was performed to repair a urinary bladder tear, along with a biopsy of the urinary bladder wall. Histopathological features consisted of lymphoplasmacytic cystitis with haemorrhages and multifocal fibrotic areas within the muscular layers. Spontaneous rupture of the urinary bladder without evidence of trauma is a well‐known, though rare, condition in human medicine. The chronic inflammation detected in the present case, along with fibrosis, caused the weakening of the urinary bladder wall, leading to perforation. This is the first documented veterinary case of spontaneous rupture of the urinary bladder secondary to chronic inflammation and highlights the importance of including this condition in the differential diagnosis of patients presenting with uroperitoneum without underlying trauma.

## INTRODUCTION

Uroabdomen (UA) is defined as the accumulation of urine within the peritoneal cavity (uroperitoneum), retroperitoneal cavity (retroperitoneum) or both, resulting from a leakage in the urinary tract. In dogs, the urinary bladder is the most common site of rupture of the urinary system, followed by the urethra (Gannon & Moses, 2003).

The condition typically presents with acute abdominal pain, signs of peritonitis and varying degrees of haematuria or urinary retention. During the initial stages, the clinical manifestations of urinary bladder rupture remain minimal, vague and diffuse, making it challenging to differentiate them from other potential causes of acute abdominal pain (Grimes et al., 2018).

The aetiology of UA differs among breeds, sex and age classes. As in humans, bladder rupture in dogs and cats most commonly results from abdominal or pelvic trauma. Less common aetiologies in veterinary medicine include urinary obstructive disorders (*e.g*. urolithiasis, feline lower urinary tract, neoplasia) and iatrogenic causes during medical procedures (Gannon & Moses, 2003; Grimes et al., 2018). If perforation of the urinary bladder occurs in the absence of external trauma or direct stimulation, it is defined as a spontaneous rupture of the urinary bladder (SRUB) (Reddy et al., 2023; Zhao et al., 2023).

A literature search was conducted across several databases (PubMed, ScienceDirect, ResearchGate, Scientific Research Publishing, Europe PMC and Google Scholar) using the following keywords: “spontaneous rupture of the urinary bladder,” “SRUB,” “spontaneous uroabdomen,” “rare causes of uroabdomen,” “lymphoplasmacytic cystitis,” “atraumatic urinary rupture” and “urinary tract rupture,” up until November 2024. No reports of SRUB have been found in veterinary medicine journals based on this search.

## CASE HISTORY

A 10‐year‐old, 40 kg, chemically castrated male mixed‐breed dog presented to the emergency service unit for acute episodes of bilious vomiting, inappetence, polydipsia and anuria. The patient was living indoors in an urban apartment, and the owner reported no trauma or previous urinary tract disorder.

On physical examination, the patient was alert but lethargic, normothermic (38.3°C), mildly dehydrated (6%) with abdominal tenderness on palpation and a positive fluid wave sign. No neurological deficits were detected.

Serum biochemical analysis revealed an elevated lactate level of 2.1 mmol/L (reference range: 0.5 to 2.0 mmol/L) and significant azotaemia, evidenced by an increased blood urea nitrogen level of 39 mg/dL (reference range: 7 to 20 mg/dL) and a creatinine level of 7.1 mg/dL (reference range: 0.5 to 1.5 mg/dL). All other parameters and blood cell counts were within the normal reference ranges.

**FIG 1:**
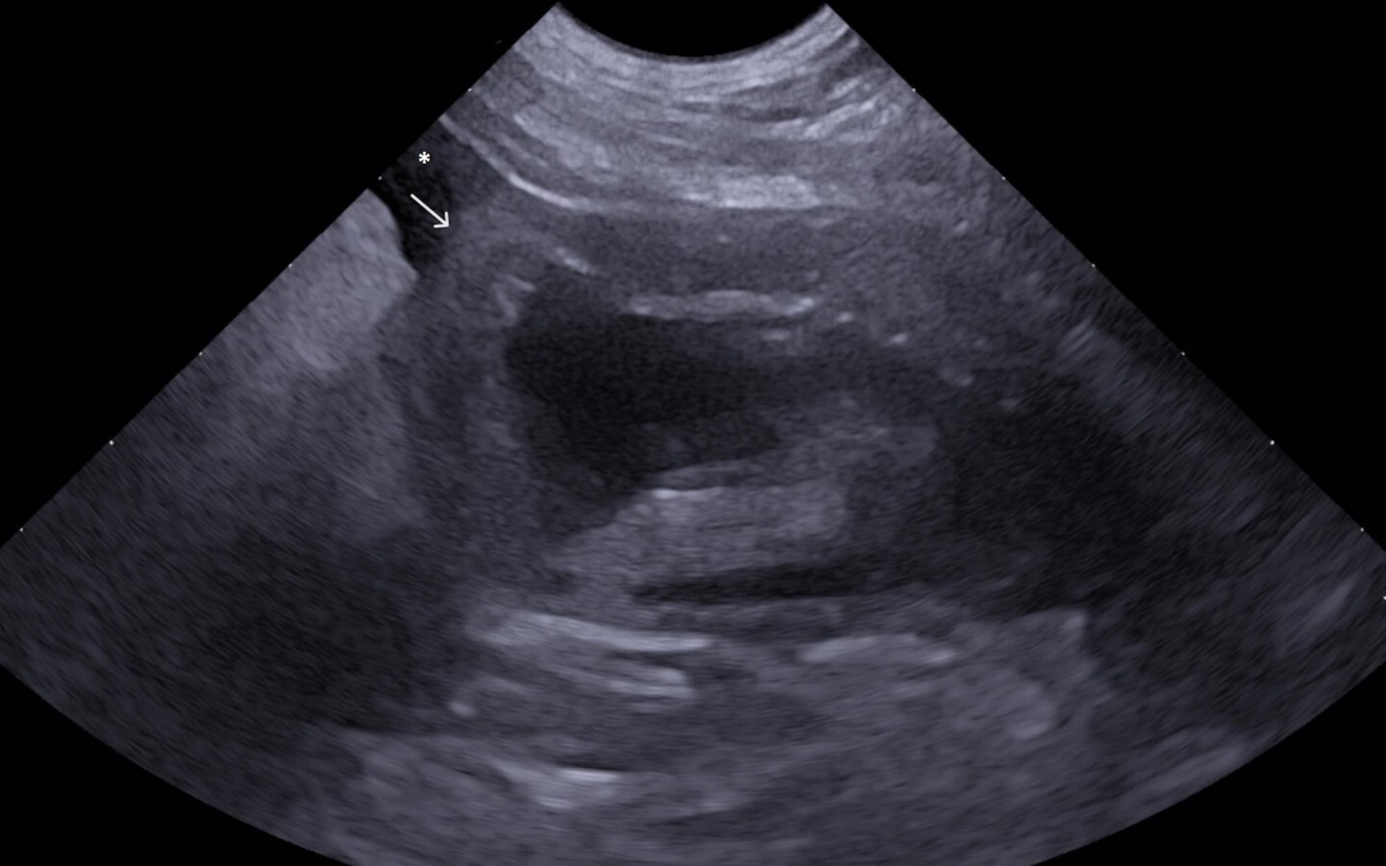
Ultrasound image obtained using a micro‐convex probe in the sagittal plane (7 MHz). The poorly distended urinary bladder has a thickened, irregular and partially mineralised wall. A focal discontinuity is noted around the apex (white arrow). The surrounding mesentery exhibits increased echogenicity and a moderate amount of anechoic effusion (white asterisk).

Ultrasonographic examination identified the presence of abdominal effusion. The exam revealed an almost empty urinary bladder surrounded by anechoic‐free fluid and hyperechoic mesentery. A mural defect was observed at the apex of the urinary bladder, which exhibited a marked thickening of approximately 1 cm. No masses or calculi were detected within the urinary bladder (Fig 1).

Ultrasound‐guided abdominocentesis and biochemical analysis of the abdominal effusion showed hyponatremia (132 mmol/L, reference range: 145 to 156 mmol/L), hyperkalaemia (7.21 mmol/L, reference range: 3.5 to 5.5 mmol/L), marked elevation of urea nitrogen level (107 mg/dL, reference range: 7 to 20 mg/dL) and creatinine level (32.24 mg/dL, reference range: 0.5 to 1.5 mg/dL). Abdominal creatinine concentration significantly exceeded the serum creatinine concentration (5.66 mg/dL, reference range: 0.5 to 1.5 mg/dL), resulting in an abdominal to serum creatinine concentration ratio>2:1.

Urinalysis collected after placement of an 8 FR Foley catheter revealed a urine specific gravity of 1.042, creatinuria (375.4 mg/dL, reference range: 0.5 to 1.5 mg/dL), urine protein‐to‐creatinine ratio of 0.27 and haematuria (>50/HPF). No leukocytes or nitrites were detected. Bacterial cell culture was not performed, as urinalysis results and the absence of clinical symptoms indicated the absence of an active urinary infection.

**FIG 2:**
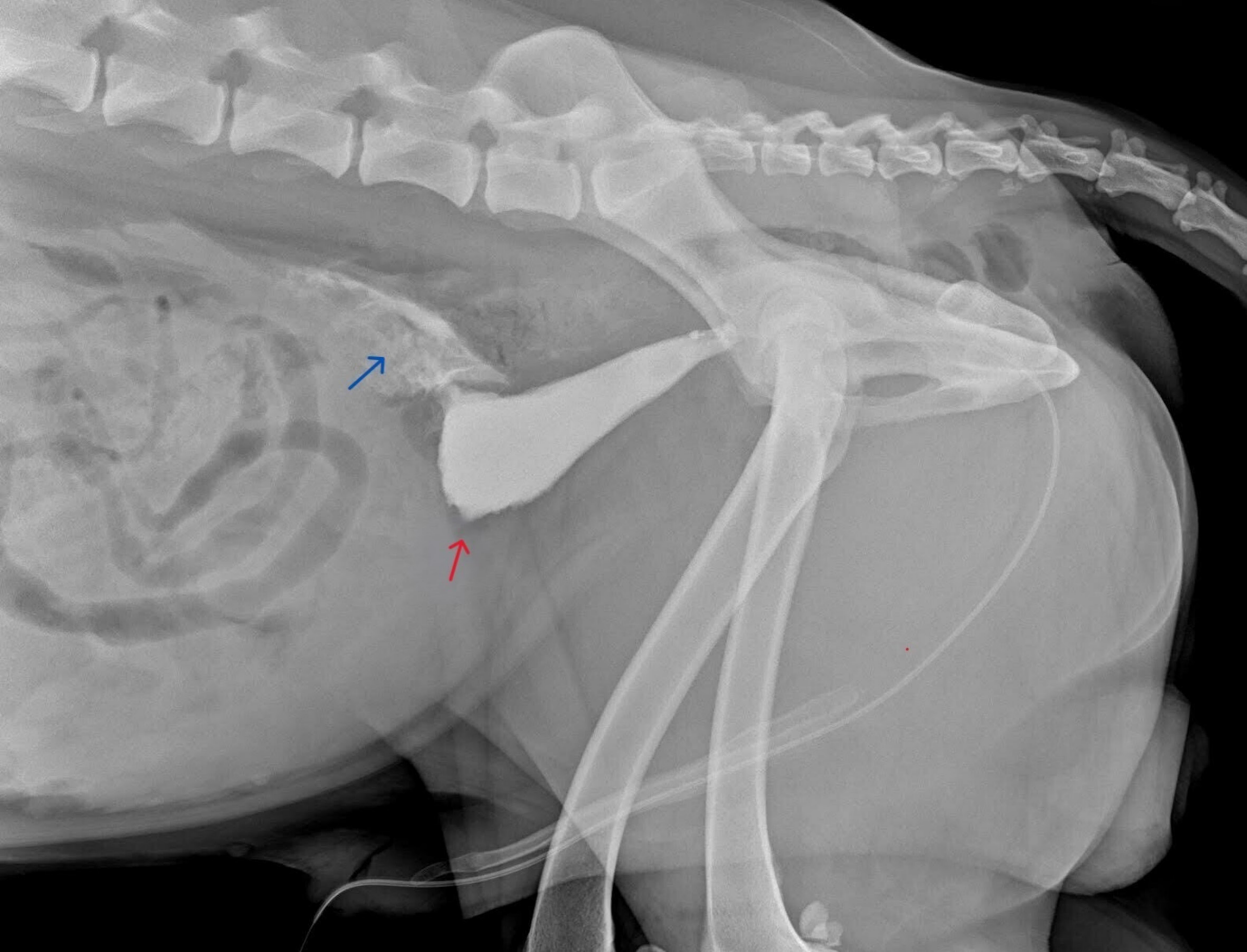
Right‐lateral radiographic urethrocystography using a positive contrast medium. There is dilation of the urinary bladder with a focal leakage of contrast medium (blue arrow) from the region of the apex (red arrow).

Plain radiographs were performed to verify the positioning of the catheter, followed by a positive contrast retrograde urethrocystography using 700 mg/kg of iodinated nonionic contrast medium (Iohexol, Omnipaque 350 mgI/mL, GE Healthcare) to confirm the presence and location of a mural lesion within the urinary bladder wall. The urinary bladder silhouette was poorly distended by contrast medium, and a focal defect was present at the apex with leakage into the caudoventral abdomen (Fig 2). These findings indicated a tear in the urinary bladder wall with a secondary uroperitoneum. The adjacent prostatic silhouette and surrounding area were normal on imaging.

**FIG 3:**
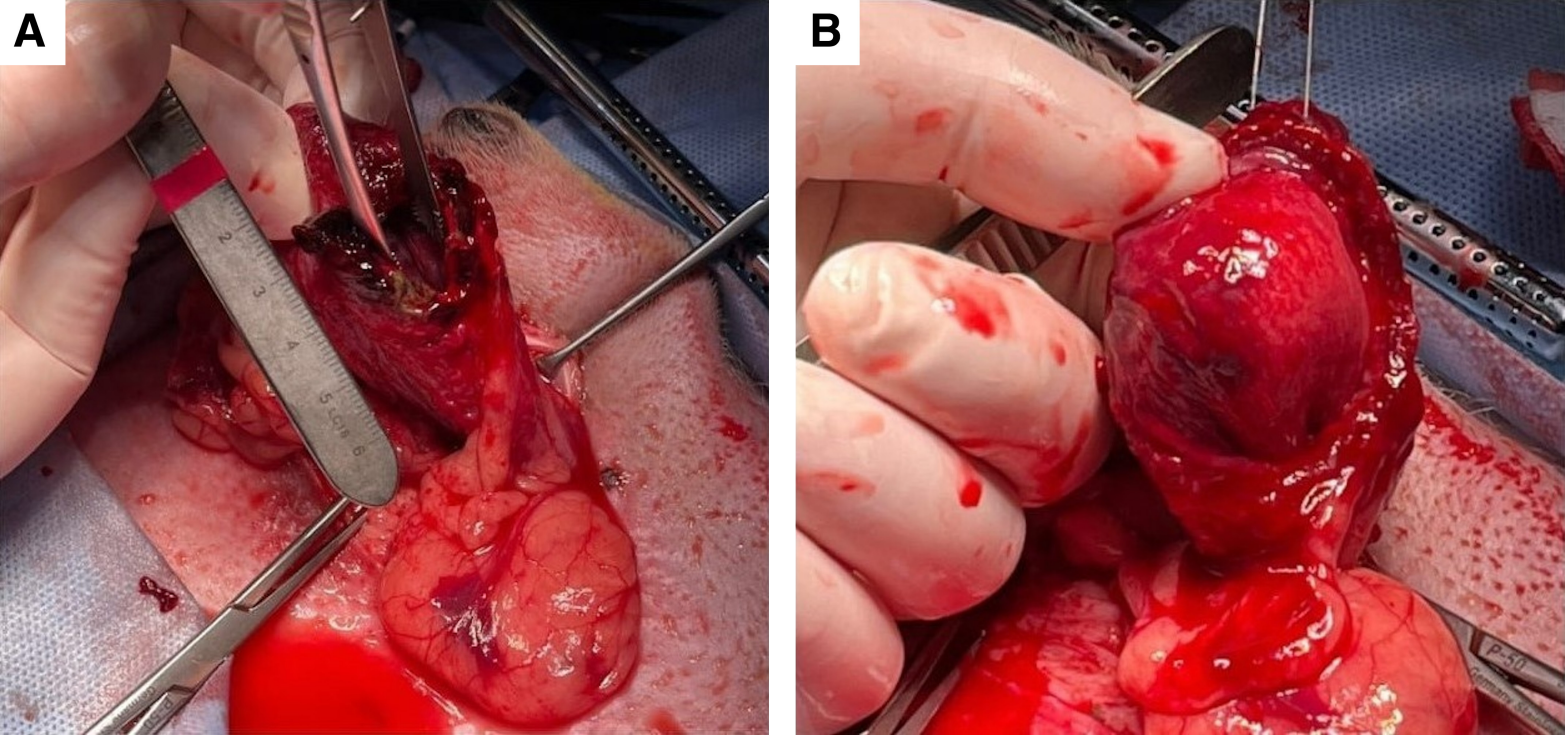
Intraoperative images showing chronic mural changes in the urinary bladder wall. Ventral‐apex urinary bladder wall tear surrounded by irregular pathological tissue, dark‐red in colour (A). Inner mucosal extension of alterations of the urinary bladder wall after surgical excision of the lesion (B).

After stabilization with intravenous balanced isotonic crystalloids at 3 mL/kg/hour for 4 hours, the patient was admitted to surgery to resolve the leakage and perform a biopsy of the urinary bladder wall to investigate possible causes of rupture. Explorative laparotomy identified a ventral‐apex urinary bladder wall tear, which was approximately 2 cm long, surrounded by grossly pathological tissue with a red‐violet colour and poorly defined margins, enveloped by well‐defined, thin and poorly vascularised adhesions on the bladder wall (Fig 3A, B). These latter changes were indicative of chronic changes resulting from prior inflammatory processes affecting the bladder wall.

The edges of the laceration were excised, including 5 mm of healthy tissue and the sample was submitted for histological examination. The defect was repaired using a non‐penetrating technique in a simple continuous pattern, followed by oversewing with a Cushing pattern, using a 2‐0 monofilament polyglycolide‐co‐caprolactone suture mounted on a taper point atraumatic needle.

A sterile Foley catheter (10 FR) was positioned for urethral catheterization. The watertight integrity of the urinary bladder sutures was verified with a gentle saline flush. The catheter cuff was then inflated with saline, secured to the abdominal wall using an anchoring system, and connected to a closed collection system.

Abundant abdominal lavage was carried out using saline solution (4 L), and the abdomen was closed in a multi‐layer fashion. Post‐surgical treatments included meloxicam (Metacam®, Boehringer) 0.1 mg/kg once a day (SID) for 5 days, gabapentin (Neurontin®, Pfizer) 10 mg/kg twice a day (BID) for 5 days, maropitant (Cerenia®, Zoetis) 1 mg/kg SID for 3 days and overnight maintenance fluid therapy with isotonic crystalloids. No antibiotics were administered. The patient remained quiet and alert with a normal temperature, pulse and respiration overnight. The urinary function was restored within a few hours, with an output production of 1.5 mL/kg/hour, urea levels of 11 mg/dL (reference range: 20 to 60 mg/dL) and creatinine levels of 1 mg/dL (reference range: <1.5 mg/dL). On the third day post‐surgery, the urinary catheter was removed, and the patient was discharged from the hospital. Ultrasound examination conducted on 2 and 7 days post‐surgery indicated an intact urinary bladder wall without evidence of leaks or omental changes around the urinary bladder apex. The patient was discharged without additional medications. One year later, the dog was reported to be in good health with no disease recurrence via a telephonic interview.

**FIG 4:**
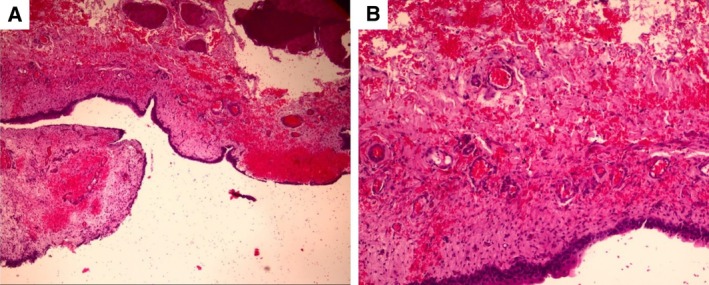
Histopathology of the urinary bladder tear at 4× (A) and 10× (B) magnification. The evaluation reveals extensive urothelial erosion and ulceration associated with wide areas of haemorrhage and marked hyperemia within the lamina propria. The muscular layer exhibits marked fibrotic deposition and detachment of muscle fibres. Mild infiltration by lymphocytes, plasma cells and neutrophils is scattered throughout the tissue sample.

Histopathological analysis of the lesion revealed erosion and ulceration in the epithelial layer associated with oedema and multifocal haemorrhages in the lamina propria. Additionally, moderate to marked deposition of connective tissue was observed in the muscular layer, where muscle fibres detached from adjacent muscular tissue. Mild infiltration of small mature lymphocytes, plasma cells and neutrophil granulocytes was also observed. The histopathological diagnosis was chronic lymphoplasmacytic cystitis with multifocal haemorrhages and focal fibrotic areas observed within the muscular compartment of the urinary bladder wall (Fig 4).

## DISCUSSION

SRUB, albeit rare, is a well‐known condition in human medicine, associated with high mortality (Zhao et al., 2023). Early diagnosis and treatment are essential for minimising morbidity and improving patient outcomes (Muneer et al., 2015). This condition results from an underlying pathology that weakens the urinary bladder wall, leading to perforation (Reddy et al., 2023; Zhao et al., 2023). In human medicine, the most common causes of SRUB are neurogenic bladder, urine retention, pelvic irradiation, invasive tumour, chronic inflammation and recurrent infection (Eghbali et al., 2022; Kivlin et al., 2015; Muneer et al., 2015; Murata et al., 2018; Rose, 1941). Recent reports described the condition as secondary to diabetes, puerperium, opioid intoxication, alcohol intoxication, haemophilia, pregnancy and emphysematous cystitis (Eghbali et al., 2022; Galbraith et al., 2011; Ranjbar et al., 2023; Rawat et al., 2017; Roels et al., 2016; Simon et al., 2023; Zhang et al., 2021). Rare cases are considered idiopathic (Reddy et al., 2023).

In veterinary medicine, bladder rupture is primarily linked to trauma but also to urolithiasis, neoplasia, urinary catheterization, aggressive bladder palpation, cystoscopy and cystocentesis (Gannon & Moses, 2003; Grimes et al., 2018). In the presented case, the urinary bladder rupture was attributed to weakness of the wall caused by underlying chronic inflammation, a less commonly reported cause in human patients.

Lymphoplasmacytic inflammation, potentially suggestive of chronic inflammation or immune system dysregulation, is characterised by the infiltration of lymphocytes and plasma cells into specific tissues, most commonly observed in the gastrointestinal tract and nasal or oral cavity (Kim et al., 2021).

Lymphoplasmacytic inflammation of the urinary tract caused by recurrent hydronephrosis and spontaneous renal rupture in a cat has already been documented (Kim et al., 2021). However, SRUB due to lymphoplasmacytic cystitis has not been previously reported in veterinary medicine.

The type of urinary bladder rupture is determined by the location of the injury and its relationship to the peritoneal reflection: the apex, being the weakest part of the bladder, is most prone to injury, making spontaneous and iatrogenic ruptures typically intraperitoneal (Reddy et al., 2023; Simon et al., 2023; Zhang et al., 2021; Zhao et al., 2023). In this instance, the rupture of the urinary bladder wall occurred around the apex due to chronic lymphoplasmacytic cystitis, secondary haemorrhages and marked focal fibrotic areas within the muscular layer. The absence of etiologic pathogens or neoplastic cells suggested that multifocal fibrotic areas observed within the muscle tissue may have caused discontinuities in the muscle fibres, contributing to the formation of less extensible regions. These structural changes likely predisposed the urinary bladder to supra‐physiological expansions, ultimately resulting in focal leaks, a mechanism extensively described in the human literature (Zhang et al., 2021).

In this context, the use of colour/power Doppler and CEUS during ultrasound examination would have been beneficial in identifying parietal ischaemia at the rupture site, providing valuable insights and further elucidating the extent of tissue damage prior to exploratory laparotomy. Such techniques should be considered in future cases when similar case presentations occur, if available.

Although in the present case, chronic inflammation was identified as the primary contributing factor to urinary bladder rupture, other potential but less likely causes should also be considered. For instance, minor trauma, such as attempting to jump onto a couch or excessive physical activity, could have contributed to SRUB already compromised by chronic inflammation and secondary fibrosis, particularly if the urinary bladder was full at the time.

In conclusion, SRUB is a rare event often presented with non‐specific clinical signs (Zhang et al., 2021). Despite its low incidence in human medicine, this condition is associated with a high mortality rate (47%), primarily due to delayed diagnosis (Reddy et al., 2023; Zhang et al., 2021). Therefore, including this pathology in the differential diagnosis is crucial for facilitating early recognition and prompt intervention, ultimately improving patient outcomes.

Based on the literature search, this is the first published case report in veterinary medicine describing an SRUB in a dog and highlights the importance of including this pathology in the differential diagnosis for patients presenting with uroperitoneum without any evidence of underlying trauma or known comorbidities.

## Author contributions

**C. Donà:** Conceptualization (lead); investigation (equal); project administration (equal); writing – original draft (lead). **M. Manfredi:** Data curation (equal); supervision (equal); validation (equal); writing – review and editing (supporting). **L. Auletta:** Data curation (equal); investigation (lead); supervision (equal); writing – review and editing (equal). **E. Brambilla:** Investigation (equal); validation (equal); writing – review and editing (equal). **J. Bassi:** Data curation (equal); investigation (equal); validation (equal); writing – review and editing (equal). **M. Zambelli:** Investigation (lead); visualization (equal); writing – review and editing (equal). **M. Longo:** Conceptualization (supporting); project administration (supporting); supervision (lead); writing – review and editing (lead).

## Conflict of interest

None of the authors of this article has a financial or personal relationship with other people or organisations that could inappropriately influence or bias the content of the paper.

## Data Availability

The authors confirm that the data supporting the findings of this study are available within the article. Raw data supporting the findings of this study are available from the corresponding author, upon reasonable request.

## References

[jsap13858-bib-0001] Eghbali, F. , Mosavari, H. , Madankan, A. , Hariri, V. , Garakani, K. & Bhahdoust, M. (2022) Generalized peritonitis secondary to spontaneous rupture of the urinary bladder in a diabetic patient: a case report. International Journal of Surgery Case Reports, 97, 107458. 35930990 10.1016/j.ijscr.2022.107458 PMC9403300

[jsap13858-bib-0002] Galbraith, J.G. , Butler, J.S. & McGreal, G.T. (2011) Opioid toxicity as a cause of spontaneous urinary bladder rupture. American Journal of Emergency Medicine, 2, 239.e1. 10.1016/j.ajem.2010.02.015 20825888

[jsap13858-bib-0003] Gannon, K.M. & Moses, L. (2003) Angell Memorial Animal Hospital – Boston, Massachusetts. Veterinaria, 17, 55–62.

[jsap13858-bib-0004] Grimes, J.A. , Fletcher, J.M. & Schmiedt, C.W. (2018) Outcomes in dogs with uroabdomen: 43 cases (2006–2015). Journal of the American Veterinary Medical Association, 252, 92–97. 29244609 10.2460/javma.252.1.92

[jsap13858-bib-0005] Kim, J. , Oh, D. , Cho, J. , Kim, S. & Yoon, J. (2021) Recurrent hydronephrosis and spontaneous renal rupture caused by lymphoplasmacytic inflammation in a cat. Veterinární Medicína, 66, 80–86. 40201715 10.17221/153/2020-VETMED PMC11975229

[jsap13858-bib-0006] Kivlin, D. , Ross, C. , Lester, K. , Metro, M. & Ginsberg, P. (2015) A case series of spontaneous rupture of the urinary bladder. Current Urology, 8, 53–56. 26195965 10.1159/000365690 PMC4483303

[jsap13858-bib-0007] Muneer, M. , Abdelrahman, H. , El‐Menyar, A. , Zarour, A. , Awad, A. & Al‐Thani, H. (2015) Spontaneous atraumatic urinary bladder rupture secondary to alcohol intoxication: a case report and review of literature. The American Journal of Case Reports, 16, 778. 26522816 10.12659/AJCR.894992 PMC4634162

[jsap13858-bib-0008] Murata, R. , Kamiizumi, Y. , Tani, Y. , Ishizuka, C. , Kashiwakura, S. , Tsuji, T. et al. (2018) Spontaneous rupture of the urinary bladder due to bacterial cystitis. Journal of Surgical Case Reports, 2018, rjy253. 30302191 10.1093/jscr/rjy253 PMC6162351

[jsap13858-bib-0009] Ranjbar, A. , Mehrnoush, V. , Montazeri, F. , Darsareh, F. , Shahrour, W. , Roozbeh, N. et al. (2023) Manifestation of spontaneous rupture of the urinary bladder in pregnancy: a systematic review of the literature. Cureus, 15, e44643. 37799223 10.7759/cureus.44643 PMC10548771

[jsap13858-bib-0010] Rawat, J. , Singh, S. & Chaubey, D. (2017) Spontaneous bladder rupture: unusual presentation in a haemophilic child. Case Reports, 2017, bcr‐2017. 10.1136/bcr-2017-220943 PMC5623995 28784897

[jsap13858-bib-0011] Reddy, D. , Laher, A.E. , Lawrentschuk, N. & Adam, A. (2023) Spontaneous (idiopathic) rupture of the urinary bladder: a systematic review of case series and reports. BJU International, 131, 660–674. 36683400 10.1111/bju.15974

[jsap13858-bib-0012] Roels, P. , Decaestecker, K. & De Visschere, P. (2016) Spontaneous bladder wall rupture due to emphysematous cystitis. Journal of the Belgian Society of Radiology, 100, 83. 30151481 10.5334/jbr-btr.1151 PMC6100414

[jsap13858-bib-0013] Rose, D.K. (1941) The urinary bladder: normal, myogenic and neurogenic. The Journal of Urology, 46, 257–270.

[jsap13858-bib-0014] Simon, L.V. , Sajjad, H. , Lopez, R.A. & Burns, B. (2023) Bladder rupture. Book of Clinical Medicine, 6, 93.

[jsap13858-bib-0015] Zhang, Y. , Yuan, S. , Alshayyah, R.W. , Liu, W. , Yu, Y. , Shen, C. et al. (2021) Spontaneous rupture of urinary bladder: two case reports and review of literature. Frontiers in Surgery, 8, 721705. 34796196 10.3389/fsurg.2021.721705 PMC8592997

[jsap13858-bib-0016] Zhao, S. , Duan, H. , Wang, Y. , Chen, H. , Wang, Y. & Li, R. (2023) Spontaneous rupture of the urinary bladder: a rare case report. Heliyon, 9, e17129. 37455977 10.1016/j.heliyon.2023.e17129 PMC10338306

